# Public Attitudes Toward Psychiatric Hospitals: A Rural-Urban Comparative Public Survey in Odisha State, India

**DOI:** 10.3389/fpsyt.2021.745604

**Published:** 2021-10-01

**Authors:** Sunita Jena, Aron Zieger, Kerem Böge, Gayatri Salunkhe, Georg Schomerus, Kripalini Patel, Bijaya Kumar Padhi, Thi Minh Tam Ta, Aditya Mungee, Eric Hahn

**Affiliations:** ^1^Asian Institute of Public Health, Utkal University, Bhubaneswar, India; ^2^Department of Psychiatry and Psychotherapy, Charité University Hospital, Berlin, Germany; ^3^Center of Medicine and Society, University of Freiburg, Freiburg, Germany; ^4^Department of Psychiatry and Psychotherapy, University of Leipzig, Leipzig, Germany; ^5^Department of Community Medicine, School of Public Health, Post Graduate Institute of Medical Education and Research, Chandigarh, India

**Keywords:** mental health stigma, psychiatric hospitals, Odisha, India, South Asia

## Abstract

**Background:** Integration of psychiatric care with public health services and offering mental health care services to patients from lower socioeconomic status remains a global challenge. Scarcity of funds and professional workforce in psychiatric hospitals contribute to this situation. However, negative attitudes in the population are also a known impediment to patients seeking mental health services. This study aimed to assess the attitudes toward psychiatric hospitals among the urban and rural population in India.

**Subjects and Methods:** The study was carried out amongst the general population in Odisha, India. The total sample (*n* = 988) includes 496 respondents from an urban-setting, and 492 respondents from rural parts of the district. Participants were selected by using simple random-sampling from the Asian Institute of Public Health (AIPH) data base. A standardized seven-item questionnaire was adopted, with responses indicated on a 5-point Likert-scale. Interviews were fully structured and conducted face-to-face.

**Results:** Level of education (*B* = −0.192, ß = −0.320, *p* < 0.000) and urban-rural comparison (*B* = −0.272, ß = −0.189, *p* < 0.000) significantly influenced attitudes toward psychiatric hospitals. Gender, age, and religious beliefs did not show any significant effect on attitudes toward psychiatric hospitals. Individuals in rural areas and those with lower education levels showed more negative attitudes toward psychiatric hospitals.

**Conclusion:** Negative attitudes toward psychiatric hospitals from those living in rural areas as well as those with lesser education may be reflective of the lack of availability, accessibility, affordability, and credibility of such mental health services. The Mental Health Care Act in India is a progressive legislation which might improve the situation of the provided services and, consequently, reduce negative attitudes in the years to come.

## Introduction

Mental health disorders have been estimated to be directly responsible for 7.4% of the disease burden globally (1). Recent evidence has shown that the burden of disease associated with mental illness has been previously underestimated and mental illness is, in fact, the leading contributor to global burden of disease regarding years lived with disability (YLDs) (2). There is a considerable imbalance between this massive disease burden and insufficient resources allocated to mental health (3). The situation is particularly calamitous in low- and middle-income countries, where an estimated 76–85% of people with severe mental health disorders receive no treatment (3). The two most populous countries of the world, India and China represent one-third of the global mental health disease burden (4, 5). However, <1% of the national health care budget is allocated to mental health services in these countries while there remains extremely low coverage of effective health care even for severe psychiatric disorders (4, 5).

Mental illness is often seen in India as a lifelong difficulty associated with a significant risk of social marginalization, health risks, and economic deprivation (6). Specifically, for India, it is estimated that 150 million people are in need of urgent treatment because of a psychiatric disorder (7). The same survey also reports a treatment gap of over 60% for all mental health disorders other than epilepsy; 80% of patients do not receive treatment 12 months after initial presentation of symptoms (7). Stigma associated to mental diseases, lack of awareness in common people, delayed treatment seeking behavior, absence of low-cost diagnostic test, and lack of easily available therapy are the primary barriers in tackling the problem of mental health in India. In addition, circumstances pertaining to traditional medicine and faith in supernatural forces in community delayed diagnosis and treatment (8). Concurrently, stigma in India often comes from mental illness illiteracy which leads to a huge number of people avoiding bio-medical therapy and instead seeking aid from traditional and religious healers (6).

In India, mental health care has still not been adequately integrated into the primary health care system (9). There are just 0.8 psychiatric hospital beds per 100,000 people in India, compared to 1.0 per 100,000 people in China, and 41.0 per 100,000 people in Germany (10). Furthermore, there is only 1 psychiatric hospital per 33 million people (10). With regards to financing, 0.6% of the total health budget in India is allocated toward mental health expenditures, compared to 11% in a developed country such as Germany (10). The first psychiatric hospitals in India under the nomenclature of “lunatic asylums” were established in the late 1700s during the British colonial rule in India (11). In the early 1900s, the nomenclature “asylums” was modified to “mental hospitals” and efforts were made to improve the standard of care (12). After India's independence in 1947, there was an increased focus on establishing psychiatric units in general hospitals. Integrating mental healthcare with general public health services and offering mental health care services to patients from lower socioeconomic status remains a challenge even in modern times (13) and psychiatric hospitals continue to play a crucial role in treating severe psychiatric disorders and patients with poor family or social support (14). Overcrowding, understaffing and inadequate government funding are some of the major problems associated with psychiatric hospital care in India (15). Taking a critical view of the situation, the National Human Rights Commission in India concluded that half of the psychiatric hospitals had exclusively closed wards; <50% hospitals had clinical psychologists and psychiatric social workers, while only 25% had qualified psychiatric nurses (15). The issue that this causes is apparent when looking at the treatment gap, and the duration of the treatment of mental disorders in India. It is worrying to 9 notes that there is a treatment gap of 73.6% for severe mental disorders such as schizophrenia (16). Furthermore, the duration of illness for those with severe mental disorders stands at 2.5 months, with a majority of the patients being treated in government facilities (16). Although there is a trend in general hospitals to create smaller psychiatric units within, larger psychiatric hospitals are still the primary mental health care delivery system in India today (12). Internationally, some studies have reported on public attitudes regarding comparisons between psychiatric hospitals and psychiatric units in general hospitals (17, 18). For instance, Angermeyer et al. reported that the image of psychiatric hospitals has improved over time in Germany, probably because of the extensive transformation of psychiatric hospitals, into smaller units (17). Similar trends have been observed in other high-income countries such as Australia and USA (19).

Considering that India has introduced a new Mental Health Care Act in 2017 (20) with a number of steps to de-stigmatize mental disorders, it is important to study whether the public perception of psychiatric hospitals in India will change over time. To our knowledge, there have been no systematic studies to investigate attitudes in the general population toward psychiatric hospitals in India, despite the fact that mental disorders and their current treatment options have been reported to be highly stigmatized (21). A study on help-seeking behavior has also shown that many patients prefer healers or non-professional treatment methods over a psychiatric hospital, perhaps because of supernatural or religious beliefs (22).

Considering these findings together with the historical perspective, we hypothesize that public attitudes toward psychiatric hospitals would be predominantly negative in India. This study aims to compare attitudes between an urban and rural population in a culturally, linguistically, and geographically similar population in India. Since most of the psychiatric hospitals are located in urban areas, we expect a higher degree of awareness and hence more positive attitudes in urban populations compared to rural populations.

## Methods

### Study Setting

We designed a community-based, cross-sectional study comparing urban and rural public attitudes toward psychiatric hospitals in the state of Odisha, India. Odisha is a state in the eastern part of India, surrounded by the states of West Bengal and Jharkhand on the North, Andhra Pradesh on the South, the Bay of Bengal on the East and Chhattisgarh on the West. Based on total population, Odisha ranks eleventh and in terms of total area it ranks ninth among all the states in India (23). The collection of data from the urban population was conducted in Bhubaneswar, the capital city of Odisha, while the rural surveys were conducted in the Khordha district. Odisha is known to have a substantial tribal population, which includes an 8.4% of the population of the Khordha district (24).

The urban city of Bhubaneswar had a population of 843,402 in 2011, of which 446,204 were male, and 397,198 were female. Of that total population, 81,847 were children between the ages of 0–6. The literacy rate in Bhubaneswar is 91.87%, and the level of employment is 95.73%. The city is known to be a fast growing hub for administration, information technology, and education. There is also a presence of large scale medical institutes such as the All India Institute of Medical Sciences, which provide the local population with good access to mental healthcare. In primary and secondary setting of this study area there is no such mental health care facility is available in this context and for this facility usually people have to depend on tertiary setting with an average distance of 8–10 km (25).

In this study, participants did not indicate their attitudes toward psychiatric hospital for particularly one health care facility, rather their attitude reflects for the all psychiatric hospitals in the region as a whole.

The rural village of Balianta in the Khordha district had a population of 3,556 in 2011, of which 1,816 were male, and 1,740 were female. Of that population, 369 were children between the ages of 0–6. The literacy rate in Balianta is 83.97%, indicating lower levels of educational attainment. The level of employment was also lower at 77.76%, indicating a lower economic status than in the urban region. With access to healthcare and psychiatric hospitals being much more restricted in this rural setting, respondents would have been indicating their attitude toward the Community Health Centre in the village (24).

### Measurement

With a lack of validated questionnaires available to measure the attitudes of people towards mental health facilities in India, we adopted the standardized seven-item questionnaire tool published by Angermeyer (26). Participants' responses were indicated on a 5-point Likert scale ranging from 1 which indicate, “definitely true” to 5 which indicate, “definitely not true.” We found three of the positively worded items needed to be reversed so that a higher score represented more negative attitudes, following which the scores for all the items were added to obtain a sum score. This sum score for each of the responses was then divided by seven resulting in a mean score ranging from one to five. Higher scores were an indication of negative attitudes, while lower scores were an indication of positive attitudes toward psychiatric hospitals.

In order to validate the Angermeyer questionnaire in the context of Odisha, at first the questionnaire was translated into the local vernacular language i.e., Odia and then back translated into English was done. Also, the tool was sent to 2 experts of public health background for their opinion and necessary changes had been made accordingly. Furthermore, context validation was done by piloting the questionnaire at the similar demographic region to our study area.

### Sample and Sampling Techniques

We used a convenient sample of 1000 participants from urban (non-slum) and rural area of Khorda district, Odisha, India. Data was collected through home visits.

### Procedure

Data was collected from August 2016 to January 2017. Informed consent was obtained prior to the data collection, with the consent form being read aloud for participants who were illiterate.

Participants were between 18 and 65 years old. Of the 1,000 total participants, 4 participants from urban settings and 8 participants from rural settings were discarded because of incomplete responses or the withdrawal of informed consent. Therefore, 496 urban participants and 492 rural participants were included in this study. Data were captured electronically.

### Ethical Approval

The study was part of the Master of Public Health (MPH) dissertation. The ethical review board of Asian Institute of Public Health, India approved the study and verbal informed consent was obtained from each participant before the interview. Respondents were made aware of the research objective and purpose of the study. Participation in this study was completely voluntary. All data was saved in an anonymized form.

### Statistical Analysis

Statistical analysis was performed by using IBM SPSS Statistics for Mac OSX, Version 21.0. Initially, items 2, 4, and 7 were reverse coded to align with the phrasing of the other items. Next, a mean score was calculated for each of the items, resulting in a value ranging from one to five, with lower scores representing positive attitudes, and higher scores representing negative attitudes. The next step involved condensing the 5-point Likert scale of all seven items to a 3-point scale to have an overview of the responses patterned “agree,” “disagree,” and “undecided” and in order to find the difference of seven items between urban and rural respondents, Chi-square test were performed with statistical significance of *p* < 0.05 (**Table 2**). Finally, a multiple linear regression (**Table 3**) was performed with the mean-score of attitudes toward psychiatric hospitals as the dependent variable and age, gender, education, strength of religious beliefs, religion, and place of living as the independent variables.

## Results

Table 1 depicted the socio-demographic profile of study participants. This study included 496 urban and 492 rural respondents between the age group 18–64 years and the mean age of respondent were 38.66 (*SD*:11.82) with 53.8% of female participants. About 52.1% were having higher education, followed by secondary education (22.78%), and primary education (25.1%). Around 68.7% of them were having strong religious belief, and 86% of the respondents were Hindu.

**Table 1:** Socio-demographics of the sample.

**Variable**	**Urban %**	**Rural %**	**Total %**
	**(*****n*** **= 496)**	**(*****n*** **= 492)**	**(*****n*** **= 988)**
**Gender**			
Male	43.1	49.2	46.2
Female	56.9	50.8	53.8
**Age (years)**			
18–24	10.5	16.7	13.6
25–34	35.5	27.6	31.6
35–44	19.8	22.0	20.8
45–54	17.5	21.1	19.3
55–64	17.7	12.6	14.7
**Education**			
Primary (1–7) or lower	3.40	47.0	25.1
Secondary (up to 10th)	9.90	35.8	22.8
Secondary or higher	86.7	17.3	52.1
**Strong religious beliefs**			
Yes	55.6	81.9	68.7
No	44.4	18.1	31.3
**Religion**			
Hinduism	87.3	86.0	86.6
Islam	3.80	11.0	7.4
Christianity	8.90	2.20	5.6
None	0.00	0.80	0.4

Table 2 shows the responses for individual items of attitudes toward psychiatric hospitals between urban and rural participants. In common three of the items had more negative responses than positive. These included: first, psychiatric hospitals have more in common with prisons than with general hospitals, second, once admitted, it is very difficult to get released from a hospital, regardless of whether the patient is ill or not, and third, psychiatric hospitals are necessary to protect society from mentally ill people. But when considering the responses of participants for all the 7 items, in comparison to urban respondents, rural respondents are having more of negative attitudes with *p* < 0.05 for all individual items.

**Table 2:** Public attitudes toward psychiatric hospitals between Urban and Rural.

**Item no**.	**Item**	**Response category**	**Sample (*****n*** **= 988)**	**Rural (*****n*** **= 496)**	**Urban (*****n*** **= 492)**	***p*** **-value**
1	Treatment in a psychiatric hospital is out of question. On the contrary, one becomes really sick there.	Agree Undecided Disagree	39.9 13.6 46.6	54.67 17.07 28.25	25.20 10.08 64.72	0.01*
2	Psychiatric hospitals are hospitals just like other hospitals, the difference being that mental illnesses and not physical illnesses are treated there	Agree Undecided Disagree	68.5 14.4 17.1	52.64 21.34 26.02	84.27 7.46 8.27	0.01*
3	Psychiatric hospitals have more in common with prisons than with hospitals	Agree Undecided Disagree	47.6 12.0 40.4	58.94 16.87 24.19	36.29 7.26 56.45	0.011*
4	Psychiatric hospitals provide the necessary security to deal with a mental crisis	Agree Undecided Disagree	69.9 14.8 15.3	57.32 22.76 19.92	82.46 6.85 10.69	0.01*
5	Once you are admitted to a psychiatric clinic, then it is very difficult to come out again, regardless of whether you are ill or not	Agree Undecided Disagree	49.9 18.2 31.9	58.74 19.31 21.95	41.13 17.14 41.73	0.015*
6	People are not treated in the psychiatric hospital but only sedated.	Agree Undecided Disagree	39.8 13.5 46.8	56.91 16.06 27.03	22.78 10.89 66.33	0.01*
7	Psychiatric hospitals are necessary to protect the society from the mentally ill patients	Agree Undecided Disagree	71.9 15.2 13.0	55.49 25.20 19.31	88.10 5.24 6.65	0.05*

*Numbers in parentheses indicate column percentages*.

* *p < 0.05*.

Table 3 displays the results of the multiple linear regression, where we analyzed the effects of gender, age, educational level, the strength of religious beliefs, religion, and place of living (urban vs. rural) as independent variables on the mean score of public attitudes toward psychiatric hospitals as a dependent variable. As reported in Table 3, level of education (*B* = −0.192, ß = −0.320, *p* < 0.000) and place of living (*B* = −0.272, ß = −0.189, *p* < 0.000) significantly influenced attitudes toward psychiatric hospitals. Gender, age, and religious beliefs did not show any significant effect on attitudes toward psychiatric hospitals. According to the mean score [Sum Score (SS)] calculated, lower education levels (SS = 3.45) meant more negative attitudes toward psychiatric hospitals compared to respondents with higher educational attainment (SS = 2.64). Similarly, it was found that respondents living in rural areas (SS = 3.24) had more negative attitudes than those living in urban areas (SS = 2.67).

**Table 3:** Public attitudes toward psychiatric hospitals: Multiple linear regression.

**Socio-demographic variable**	**Survey (%)** **(*****n*** **= 988)**	**Sum of items 1 through 7 divided by 7 Mean [SD]**	***p*** **-value**
**Gender**			
Male	46.2	2.95 [0.68]	n.s.
Female	53.8	2.96 [0.75]	
Age, years: Mean (SD)		38.66 [11.83]	
18–24	13.6	2.82 [0.74]	
25–34	31.6	2.89 [0.66]	
35–44	20.9	2.98 [0.72]	n.s.
45–54	19.3	3.12 [0.72]	
55–64	14.7	2.95 [0.79]	
**Educational level**			
Primary school or lower	25.1	3.45 [0.67]	
Middle school	22.8	3.14 [0.71]	*p* < 0.000
Secondary school or more	52.1	2.64 [0.58]	
**Strong religious beliefs**			
Yes	68.7	3.03 [0.73]	n.s.
No	31.3	2.80 [0.67]	
**Religion**			
Hinduism	86.6	2.96 [0.72]	
Islam	7.40	3.29 [0.57]	n.s.
Christianity	5.60	2.57 [0.65]	
None	0.40	1.46 [0.32]	
**Place living**			
Urban	50.2	2.67 [0.72]	*p* < 0.000
Rural	49.8	3.24 [0.60]	

*n.s., not significant*.

To further explore these results, we conducted a follow up ANOVA with place of living as the fixed factor and the sum score for attitudes toward psychiatric hospitals as the dependent factor. The results [*F*_(1,986)_ = 183.65, *p* < 0.001] confirmed that the area in which the individual lived had a highly significant effect on attitudes toward psychiatric hospitals. Since 86.7% of our urban population had completed secondary school education as opposed to only 17.3% in the rural population, it raises an important question if the urban population has lesser negative attitudes simply because of the higher education levels. Hence, we conducted a follow up ANCOVA with education as a covariate and place of living as a fixed factor. Even after including education as a covariate, we found a significant effect for place of living [*F*_(1,985)_ = 12.700, *p* < 0.001] indicating that in spite of controlling for education as a confounding factor, other unidentified factors contributed to the significantly higher negative attitudes in the rural population. Additionally, we studied if highly educated people show difference in their attitudes depending on their place of living. An independent samples *t*-test was also performed as a follow-up with place of living as the grouping variable and the sum score as the test variable. The results indicated that highly educated individuals in a rural setting (*M* = 2.90, *SD* = 0.72) showed more negative attitudes than highly educated in urban settings (*M* = 2.59, *SD* = 0.53); *t*_(4.69)_ = 513, *p* < 0.000.

## Discussion

Various studies were conducted with revealed that attitude toward psychiatrist (27), mental health care (25). However, to our knowledge this is the first ever study which explored the attitudes of rural people toward psychiatric hospital. The primary aim of this research study was to investigate public attitudes toward psychiatric hospitals in the eastern province of Odisha, India, a region with little existing research data having struggled with marginalized and socioeconomically struggling communities. More specifically, the aim was to investigate attitudes of individuals living in rural areas compared to people living in urban areas. Additionally, other sociodemographic variables including gender, age, education, strength of religious beliefs, religion were analyzed with multiple linear regression on mean score of a seven-item questionnaire for attitudes toward psychiatric hospitals. There were some interesting observations to be made when looking at the data as a whole. For example, nearly half of the participants found that psychiatric hospitals were more comparable to prisons than regular hospitals. Furthermore, nearly 40% of the participants found that patients only get sicker in psychiatric hospitals, or are simply sedated, rather than being treated. The results showed an association of more negative attitudes of respondents living in rural areas compared to those with less education. Interestingly, place of living significantly influenced attitudes toward psychiatric hospitals even after controlling for education. Highly educated rural individuals showed significantly higher negative attitudes when compared to highly educate urban individuals. Other sociodemographic variables did not significantly affect negative attitudes toward psychiatric hospitals. Contrast to our findings, a study conducted in India was aimed to see if there are any differences in treatment seeking between rural and urban patients with psychosis. Patients in metropolitan regions do not seek psychiatric care as early as patients in rural areas, despite being closer to psychiatric centers, having a higher level of education, and having a higher income. As a result, simply having services available does not guarantee that patients will use them (28).

Previous studies from India and other developing countries have also found an association between mental health stigma and rural populations as well as lower education (29–31). Educational attainment and literacy of a population have often been reported as key factors when discussing issues within the mental health system (32). Lower literacy rates are associated with a lack of mental health literacy, especially regarding acknowledging mental illness, or seeking knowledge about adequate treatment, which could involve admittance to psychiatric hospitals (29, 33, 34). While the proportion of the population in India that is both, seeking and attaining education, literacy is constantly rising, even in rural areas where the proportion of persons without formal education is still relatively high compared to other high population middle income countries, such as China (35).

In India, nearly 70% of the population still lives in rural areas (24), and that population usually has less access to education in comparison to those living in urban areas (35). As hypothesized, we found more negative attitudes toward psychiatric hospitals from respondents living in rural areas. This is in line with a previous study conducted in India (36), as well as in other low- and middle-income countries (37). In addition to a relative lack of educational attainment in rural areas associated with negative attitudes, rural communities are more often confronted with a scarcity of resources, limited access to healthcare, and reliance on traditional belief systems (38).

To see if there are any differences in treatment seeking between rural and urban patients with psychosis, patients in metropolitan regions do not seek psychiatric care as early as patients in rural areas, despite being closer to psychiatric centers, having a higher level of education, and having a higher income. As a result, simply having services available does not guarantee that patients will use them (28). Similarly, another study stated anticipated stigma from community and healthcare provider were the reason for delays in seeking help and discontinuation of treatment (39). Furthermore, Lahariya et al. (22) highlighted that mentally ill patients do not seek help from any health facility because of certain reasons such as lack of awareness about the services related to treatment, distance of the health facility and due to fear of stigma associated with treatment.

It has been reported that while populations living in rural communities are comparatively accepting of people who live with mental illness in their communities, they often share beliefs that direct their negative attitudes toward psychiatry and psychiatric treatment options (30). When researchers presented to people living in rural settings vignettes depicting a person suffering from psychosis or depression, a large majority did not acknowledge this person of having an illness in a medical sense while further symptoms of psychosis were perceived more frequently as dangerous (30).

While progress has been made in recent years with the introduction of the Mental Healthcare Act 2017, with provision to empower accessibility to mental health services for all, a lack of facilities and infrastructure provided by the government in rural areas has been a major part of the current problematic situation (40). This goes a long way toward explaining why a majority of the participants in this study responded that psychiatric hospitals have more in common with prisons than with regular hospitals, or that once admitted as a patient, it is very difficult to come out regardless of how severely ill one is. It is known that the condition of many public hospitals in India is appalling while an extreme lack of infrastructural capabilities ties in with a lack of power or decision-making abilities by the patients (41–43). Despite the introduction of several policy and programmatic interventions over the past three decades, there is still only roughly one trained psychiatrist for every 250,000 people in India, and less than one of any sort of mental healthcare professional (psychiatrists, psychologists, psychiatric social workers) for every 100,000 people (40). This means that treatment is unavailable or inaccessible even for those who actively seek it, which leaves the country still far from the goal of being able to provide universal health coverage (44). The Mental Healthcare Act passed in 2017 proposes an ambitious plan to improve the national mental healthcare system in the coming years, with one of the goals being to achieve the desired universal mental health coverage (45). While the act is wide-ranging and progressive in many ways, some of the key aspects that could have a direct influence on improving attitudes toward psychiatric hospitals include plans to not only improve allocated funding, but also to further establish healthcare facilities in general hospitals, as well as promote mental health education and prevention programmes (45). Furthermore, the decriminalization of suicide as well as the assurance of free treatment for those who are homeless or living below the poverty line works toward providing the right to mental healthcare access to everyone from mental health services run or funded by the government (45). Promoting healthcare education in rural areas as well the adequate training and funding of healthcare workers would also be necessary steps (40). In that regard, studies conducted in countries such as Germany (41) did report that improvement of attitudes toward psychiatric hospitals are possible if psychiatric reforms are undertaken, now actions aimed at reforming psychiatric hospitals in India must be implemented. For community literacy in India, two major things should be considered. The first is to provide myth-busting interventions to rectify incorrect beliefs in the community that may be negatively affecting treatment. The second is highlighting mental disease recovery and illustrating how healthcare practitioners play an important role in that process (46).

There were several limitations to this study. For one, it is difficult to generalize the results of this study with 988 respondents to the whole country which has a population of over 1.3 billion. Furthermore, this study only looked at one region of India, a country known to be culturally and socially diverse. Further studies could expand this research topic by including other cities and states, while including a greater sample size as well as exploring the current availability, accessibility, affordability and quality of services provided by MH institutions to the general public. Finally, this study only analyzed attitudes toward psychiatric hospitals against a few variables. This leaves the possibility for other confounding variables we did not include in the study which could have an effect on the results, such as the participant's familiarity with psychiatric illness and psychiatric hospitals.

## Conclusion

This study compared the attitudes toward psychiatric hospitals of respondents living in urban and rural Odisha, while considering the influence of other sociodemographic factors. Lesser educational levels as well as living in rural settings were associated with more negative attitudes toward psychiatric hospitals. In the future, it would be beneficial to assess a wider range of sociodemographic factors, include larger areas of India and analyse changes of attitudes over time. Furthermore, a wider scope of insights could be gained conducting studies that include qualitative measures. While progress is gradually being made, especially with the introduction of the Mental Healthcare Act 2017, there is much room to implement these ambitious steps supported by the Act into public mental healthcare in India (32).

## Data Availability Statement

The raw data supporting the conclusions of this article will be made available by the first and corresponding authors, without undue reservation.

## Ethics Statement

The studies involving human participants were reviewed and approved by Asian Institute of Public Health, Odisha, India. The patients/participants provided their verbal informed consent to participate in this study.

## Author Contributions

SJ and KP led the development of the tool. SJ conceived the manuscript, conducted the analysis, and led the writing. AZ, KB, GSa, GSc, BKP, TT, AM, and EH supported the development of the tool and the writing of the manuscript. All authors read and approved the final manuscript.

## Conflict of Interest

The authors declare that the research was conducted in the absence of any commercial or financial relationships that could be construed as a potential conflict of interest.

## Publisher's Note

All claims expressed in this article are solely those of the authors and do not necessarily represent those of their affiliated organizations, or those of the publisher, the editors and the reviewers. Any product that may be evaluated in this article, or claim that may be made by its manufacturer, is not guaranteed or endorsed by the publisher.
